# Coaching at the sport social work boundary: a competency-informed perspective for athlete well-being

**DOI:** 10.3389/fspor.2026.1918449

**Published:** 2026-08-20

**Authors:** Matt A. Moore, Lawrence W. Judge, Jerry Reynolds, Kim Taylor

**Affiliations:** 1 College of Social Work, University of Kentucky, Lexington, KY, United States; 2 Marieb College of Health and Human Services, Florida Gulf Coast University, Fort Myers, FL, United States; 3 Department of Social Work, Ball State University, Muncie, IN, United States

**Keywords:** athlete well-being, coach education, coaching, ethical practice, interprofessional collaboration, sport social work

## Abstract

Coaching has expanded beyond technical instruction into developmental leadership that includes athlete welfare, team culture, ethical decision-making, and long-term well-being. This expanded role creates important overlap with sport social work, particularly around person-in-environment thinking, cultural humility, trauma-informed practice, psychosocial risk recognition, and interprofessional collaboration. That overlap, however, must not be mistaken for role substitution. Coaches are not social workers, and sport social workers are not auxiliary performance staff. This perspective advances a competency-informed framework for connecting coaching and sport social work while preserving professional scope. The framework is organized around five domains: athlete-centered development, ethical and reflective practice, culturally responsive coaching, psychosocial risk recognition and referral, and systems-level collaboration. The central argument is intentionally bounded: coaches can become more social work-informed without becoming social workers. Coaches can create safer and more responsive sport environments, recognize when athlete needs exceed the coaching role, and activate qualified support. Sport social workers provide specialized expertise in assessment, intervention, advocacy, policy development, and systems change. The article concludes with implications for coach education, sport social work training, organizational policy, and research.

## Introduction

Coaching has expanded from a primarily performance-centered role to a complex form of developmental leadership (1, 2). Contemporary coaches teach sport skills, shape team culture, support motivation, communicate with stakeholders, respond to psychosocial concerns, and protect athlete welfare. This broader role aligns with sport social work commitments to holistic development, ethical practice, person-in-environment assessment, social justice, and systems-level well-being (3, 4). Sport social work promotes social justice and social change by focusing on the unique needs of athletes at both an individual and environmental level. Sport social workers promote health and well-being of athletes through direct practice, community organizing, advocacy, policy development, education, and research (4). Currently, sport social workers can be found across the sport ecosystem, including schools and universities, professional and amateur athletic organizations, community and youth sport programs, and sport governing bodies (5). By partnering with coaches, sport social workers might strengthen athlete well-being, team functioning, and the overall culture of sport (4).

This alignment has practical value only when it is defined with precision. Shared concern for athlete well-being is not enough. A defensible perspective must specify each profession's contribution, identify shared responsibilities, preserve boundaries, and describe the organizational mechanisms needed to translate shared values into practice. Without that structure, holistic coaching risks becoming aspirational rather than accountable.

This perspective advances a competency-informed approach for integrating sport social work principles into coaching without collapsing professional roles. These types of competency-informed approaches exist for sport psychologists, athletic trainers, and other professionals working within athletic spaces (6, 7). Five linked domains provide a practical pathway for coach education programs, athletic departments, clubs, and community sport organizations seeking to strengthen athlete well-being while preserving professional scope.

The contribution of this perspective is not the general claim that coaches should care about athlete well-being; that position is already embedded in much contemporary coach education. Its contribution is a boundary and implementation framework that distinguishes social work-informed coaching from social work practice. By separating awareness, relational response, referral, and systems collaboration from clinical assessment and intervention, the framework offers a more accountable way to prepare coaches, guide organizations, and protect athlete welfare.

## Why sport social work matters for coaching

Sport is often framed as a setting for character development, belonging, and growth. Yet sport can also reproduce exclusion, silence distress, normalize harmful power dynamics, and prioritize performance over long-term health (8). Sport social work matters because it brings disciplined attention to the social conditions that shape athlete experiences, including family systems, identity, poverty, trauma, disability, race, gender, organizational culture, and access to resources (4).

For coaches, this perspective matters because athletes do not enter practice as isolated performers. They bring histories, relationships, stressors, motivations, and identities that affect learning and performance (9). Contextually aware coaches can build trust, communicate clearly, avoid deficit-based interpretations, and recognize when athlete needs exceed the coaching role (10). This does not make coaches clinicians; it makes them safer observers, responders, and connectors to qualified support.

Sport social workers can strengthen this infrastructure by helping teams and organizations develop policies, referral pathways, prevention programs, and training that address athlete welfare before concerns escalate (5). Their contribution is not limited to crisis response; it includes education, assessment, advocacy, consultation, care coordination, and systems change. This work could also be interprofessional in nature by working closely with sport psychologists, athletic trainers, sport administrators, and other members of the sport environment (11).

## A competency-informed perspective

The proposed perspective translates shared coaching and sport social work values into five practice domains. These domains do not replace existing coaching standards; they provide a social work-informed lens for coach education, supervision, and organizational accountability. The framework also clarifies a central implementation principle: coaches should be prepared to notice, respond, and refer, while sport social workers and other qualified professionals such as sport psychologists retain responsibility for specialized assessment, intervention, advocacy, and care coordination (Table 1).

**Table 1:** A sport social work-informed coaching perspective.

Domain	Coaching application	Sport social work contribution	Example indicators
Athlete-centered development	Build autonomy, competence, belonging, and life-skill transfer.	Frame athletes as whole persons within social and environmental contexts.	Athlete voice; goal alignment; retention; perceived support.
Ethical and reflective practice	Use reflection, feedback, and supervision to examine power and decision-making.	Apply ethical reasoning, boundaries, dignity, and client-centered values.	Documented reflection; clear team norms; transparent discipline.
Culturally responsive coaching	Adapt communication and support to athlete identity, culture, disability, and lived experience.	Strengthen cultural humility, inclusion, advocacy, and anti-oppressive practice.	Inclusive policies; athlete belonging; reduced participation barriers.
Risk recognition and referral	Recognize warning signs and connect athletes to qualified support.	Provide assessment, clinical referral, crisis response, and care coordination.	Referral protocol; mental health literacy; safeguarding response time.
Systems-level collaboration	Work with families, athletic trainers, counselors, administrators, and community partners.	Address structural barriers and coordinate interdisciplinary support.	Interprofessional meetings; resource maps; policy revisions.

### Domain 1: athlete-centered development

Athlete-centered coaching emphasizes development of the person, not only the performer. This includes technical growth, confidence, autonomy, resilience, communication, and the transfer of lessons from sport to other life domains (12, 13). The strength of this domain is that it treats performance and well-being as connected priorities rather than competing aims.

Sport social work deepens this domain through the person-in-environment perspective (4). Coaches may observe effort, behavior, and performance, but those observations remain incomplete without attention to context. Absences, emotional withdrawal, conflict, or inconsistent performance may reflect issues beyond motivation or discipline [e.g., family stress, mental health concerns, interpersonal difficulties, and challenges adjusting to various athletic and life demands; (14)]. A social work-informed coach asks better questions, avoids premature judgment, and involves additional support when needed.

### Domain 2: ethical and reflective practice

Coaching is inherently relational and therefore ethically complex. Coaches influence playing time, feedback, selection, access, discipline, sportsmanship, player care (e.g., playing with injury and playing players through injury), tactics, toxic culture, and the emotional climate of the team. These power dynamics require more than good intentions; they require structured reflection, role clarity, and accountability (15).

Reflective practice is a critical bridge between coaching and sport social work. Coaches should be trained to examine how decisions affect athlete dignity, safety, fairness, and voice (16, 17). Reflection should move beyond informal self-assessment and become part of coach development through mentoring, supervision, peer consultation, and documented learning (14). This approach shifts ethics from occasional compliance to embedded professional practice and increased professionalism (18).

### Domain 3: culturally responsive coaching

A rigorous sport social work lens requires attention to culture, identity, and access. Athletes differ by race, ethnicity, gender, disability, socioeconomic status, religion, sexuality, language, immigration status, family structure, and prior sport experiences (19–21). These differences shape how athletes experience authority, belonging, pressure, feedback, and safety.

Culturally responsive coaching is not a generic commitment to being positive. It requires awareness of bias, adaptive communication, avoidance of deficit-based explanations, and team norms that protect dignity (16). Sport social workers can help organizations assess barriers to participation, build inclusive policies, and respond when team cultures become exclusionary or harmful (4, 22).

### Domain 4: psychosocial risk recognition and referral

Coaches are often among the first adults to notice changes in athlete behavior. They may observe withdrawal, sudden performance decline, fatigue, conflict, disordered eating behaviors, injury-related distress, family instability, or signs of maltreatment. Coaches need sufficient training to recognize concern, respond supportively, and activate referral pathways (23–25). They do not need, and should not claim, clinical competence beyond their training.

This boundary is central to the model. A sport social work-informed coach should notice, listen, document when appropriate, reduce immediate harm, and refer. The coach should not diagnose, provide therapy, promise confidentiality that cannot be maintained, or manage crises alone (4). Organizations should provide clear protocols for mental health concerns, abuse reporting, emergency response, documentation, and communication with families and professionals (26).

### Domain 5: systems-level collaboration

Athlete well-being is shaped by systems, not only individual choices. Team rules, travel demands, selection policies, scholarship pressure, academic expectations, medical access, family resources, and organizational culture all influence athlete development. Coaches are important actors in these systems, but they cannot carry the full burden of athlete welfare alone.

A stronger model positions coaches within an interdisciplinary network that may include sport social workers, sport psychologists, athletic trainers, mental health clinicians, strength and conditioning professionals, academic support staff, disability services, administrators, families, and community agencies (11, 27). The practical question is not whether coaches should care about well-being, but whether the sport organization has built the infrastructure to support it.

## Discussion: implications for education, practice, and research

For coach education, the immediate implication is that holistic development must be translated into applied competencies. Training should include case-based ethical decision-making (11), trauma-informed communication (22), cultural humility (16), referral protocols, mandated reporting, professional boundaries (26), and interdisciplinary collaboration (11). Coaches should rehearse responses to athlete distress, parent concerns, teammate exclusion, and conflicts between performance demands and athlete welfare.

Assessment should also change. This shift is necessary because traditional performance metrics often overlook the broader conditions that shape athlete well-being, development, and long-term participation (4). In this context, organizations should use a more comprehensive assessment approach that holds coaches and programs accountable not only for competitive outcomes but also for creating environments that promote safety, belonging, support, and holistic development. If holistic development is a stated aim, organizations should evaluate more than wins, technical improvement, or athlete satisfaction. Potential indicators include retention, athlete sense of belonging, perceived coach support, referral completion, injury communication climate, team norms, family engagement, and documented professional development (28, 29). These measures make athlete well-being visible and accountable.

For sport social work, the implication is equally important. The field can contribute to coaching contexts through training design, consultation, crisis response, care coordination, policy review, advocacy, and evaluation (5, 30–32). However, sport social work must also define its own boundaries and competencies. This can be achieved by establishing clear standards of practice, defining the knowledge and skills required for sport social workers, and identifying specific roles and responsibilities that distinguish the profession from coaching, athletic training, sport psychology, and other disciplines (4, 31). The field should avoid becoming an undefined label for any helping activity in sport. Its value rests in professional preparation, ethical standards, systems thinking, advocacy, and evidence-informed practice (Table 2).

**Table 2:** Boundary-to-action guide for coaches.

Coach responsibility	Boundary that must remain	Organizational requirement
Notice behavior, mood, participation, or safety changes.	Do not diagnose or interpret symptoms as clinical findings.	Train coaches in warning signs and documentation.
Listen with calm, nonjudgmental support.	Do not provide therapy or guarantee absolute confidentiality.	Provide scripts, referral contacts, and reporting guidance.
Activate referral or emergency protocols when risk is present.	Do not manage crisis situations alone.	Maintain referral pathways and crisis procedures.
Join care coordination when appropriate.	Do not request or share protected information beyond role need.	Clarify information-sharing and communication rules.

## Implementation and accountability

Implementation should be judged by organizational practice, not by aspirational language. A social work-informed coaching environment requires written referral pathways, role-specific training, documentation expectations, information-sharing rules, and periodic review of whether policies are used when concerns arise. These procedures make the framework auditable: they clarify who responds, who refers, who coordinates care, and how athlete privacy and safety are protected.

This accountability emphasis is important because role confusion can create significant risks to athlete safety, well-being, and ethical care (11). When coaches attempt to manage psychosocial concerns without referral pathways, athletes may receive inconsistent support, privacy expectations may become unclear, and serious concerns may remain inside the team rather than moving to qualified care. A stronger model therefore places responsibility at both levels: coaches need competencies appropriate to their role, and organizations need systems that make appropriate action possible.

## Future research directions

Future research should examine how social work-informed coach education affects athlete outcomes, coach behavior, and organizational culture. Useful designs include intervention studies, mixed-methods evaluations, longitudinal studies, and case studies of interdisciplinary support models. Researchers should also examine implementation barriers, including time, resistance, limited resources, confidentiality, and boundary uncertainty.

A second priority is measurement. The field needs tools that capture whether coaches create environments that are psychologically safe, inclusive, ethical, and developmentally responsive. Measures should be tested across youth, scholastic, collegiate, adaptive, professional, and community sport. A stronger evidence base would move the conversation from conceptual alignment to demonstrable impact.

## Conclusion

The evolution of coaching creates a meaningful opportunity for sport social work, but the opportunity must be framed with rigor. Coaches are central to athlete development, yet they should not be positioned as substitute social workers or informal clinicians. A defensible model recognizes coaches as frontline developmental leaders who can apply social work-informed principles while relying on trained professionals for specialized assessment, intervention, advocacy, and systems change.

This perspective strengthens the connection between coaching and sport social work by emphasizing competencies, boundaries, referral pathways, cultural responsiveness, and organizational accountability. The result is a clearer model: coaches remain coaches, sport social workers remain specialized professionals, and sport organizations better support performance, dignity, belonging, and long-term well-being.

## Data Availability

The original contributions presented in the study are included in the article/Supplementary Material, further inquiries can be directed to the corresponding author.
